# Electro-optic comb based real time ultra-high sensitivity phase noise measurement system for high frequency microwaves

**DOI:** 10.1038/s41598-017-03049-5

**Published:** 2017-06-06

**Authors:** N. Kuse, M. E. Fermann

**Affiliations:** 1 IMRA America Inc., Boulder Research Labs, 1551 South Sunset St, Suite C, Longmont, CO 80501 USA; 2 IMRA America Inc., 1044 Woodridge Ave, Ann Arbor, MI 48105 USA

## Abstract

Recent progress in ultra low phase noise microwave generation indispensably depends on ultra low phase noise characterization systems. However, achieving high sensitivity currently relies on time consuming averaging via cross correlation, which sometimes even underestimates phase noise because of residual correlations. Moreover, extending high sensitivity phase noise measurements to microwaves beyond 10 GHz is very difficult because of the lack of suitable high frequency microwave components. In this work, we introduce a delayed self-heterodyne method in conjunction with sensitivity enhancement via the use of higher order comb modes from an electro-optic comb for ultra-high sensitivity phase noise measurements. The method obviates the need for any high frequency RF components and has a frequency measurement range limited only by the bandwidth (100 GHz) of current electro-optic modulators. The estimated noise floor is as low as −133 dBc/Hz, −155 dBc/Hz, −170 dBc/Hz and −171 dBc/Hz without cross correlation at 1 kHz, 10 kHz, 100 kHz and 1 MHz Fourier offset frequency for a 10 GHz carrier, respectively. Moreover, since no cross correlation is necessary, RF oscillator phase noise can be directly suppressed via feedback up to 100 kHz frequency offset.

## Introduction

Microwave photonics^1, 2^ is a rapidly expanding field for handling the explosive growth of data rates as found in broadband wireless communication, radar, satellite communication, and electric warfare systems, greatly improving the operational frequency and bandwidth. Since spectral purity, i.e. phase noise, of microwaves sets the ultimate performance limits of RF links, great efforts have been expended for low noise microwave generation, including methods based on optoelectronic oscillators (OEOs)^3, 4^, optical frequency division (OFD)^5–7^, electro-optic (EO) combs^8–10^, Kerr combs^11^, and Brillouin lasers^12, 13^. Among them, OFD incorporating ultra-low noise frequency combs has resulted in extremely spectrally pure microwaves at a 12 GHz carrier frequency, achieving a phase noise <−170 dBc/Hz, although the carrier frequency is limited to harmonics of the repetition frequency of the comb^7^.

Actively suppressing phase noise of RF oscillators is a more general way for the generation of low noise RF microwaves at arbitrary carrier frequencies^9, 10^. For this, real time high sensitivity measurement of phase noise is critical, since the measurement sensitivity sets the ultimate achievable phase noise. Real time high sensitive phase noise measurement systems can also simplify the characterization of ultra-low phase noise signals, eliminating the need for the cross correlation method^14^. Data acquisition times for phase noise estimates can be greatly reduced and the issue with possible underestimation of phase noise can be eliminated.

Conventionally, phase noise is characterized via mixing of two microwaves signals^15^, regardless of whether cross correlation is used or not. In addition to the device under test (DUT), a second low noise microwave reference source is required, which need to be synchronized to the DUT. After mixing, the phase noise gets down-converted to DC and can be measured by a signal analyzer. The phase noise of the microwave reference should further be lower than that of the DUT to enable meaningful measurements. These two above constraints are generally difficult to satisfy without extensive experimental complexity. To overcome these difficulties, a photonics-based delayed self-homodyne technique can be used^16^. In the photonics-based delayed self-homodyne technique, a photonic delay is used as a frequency discriminator instead of an RF delay line to minimize propagation loss and sensitivity to electromagnetic interference. The sensitivity of the self-homodyne method can reach about −145 dBc/Hz, −153 dBc/Hz, and −158 dBc/Hz for a 10 GHz carrier at 10 kHz, 100 kHz, and 1 MHz Fourier frequency offset, respectively, without implementation of cross correlation. For both methods, all components which are used for O/E or E/O conversion, RF amplifiers, RF phase shifters, RF isolators, and RF mixers etc, need to have a bandwidth larger than that of the DUT frequency. This is a serious impediment to phase noise measurements at high microwave frequencies. Very recently, a photonic down-conversion technique without the need for a large bandwidth photo detector and RF mixer has been demonstrated, in which two electro-optic modulators (EOMs) are used, and a photonic delay is installed between the two EOMs^17^. In this method, because the DUT frequency is downconverted to DC in the optical domain, not via an RF mixer, it does not rely on large bandwidth components. Moreover, the sensitivity of the method is independent of the DUT frequency unless it reaches the fundamental limit from delay fiber noise at the DUT frequency. However, the demonstrated sensitivity of −137 dBc/Hz at 10 kHz frequency offset for a 10 GHz carrier, which is probably limited by background noise such as electronic and shot noise, has not been as good as achievable with the photonics-based delayed self-homodyne method.

In this letter, we propose and demonstrate a technique for real time, high sensitivity phase noise measurement, i.e. without necessarily relying on cross correlation, which can be extended to high frequencies. Since we exploit self-heterodyning as well as sensitivity enhancement via the use of high-order sidemodes from an EO comb, both high sensitivity and high frequency capability can be retained simultaneously. In a proof of concept experiment, we demonstrate −155 dBc/Hz, −170 dBc/Hz and −171 dBc/Hz sensitivity for a 10 GHz carrier at 10 kHz, 100 kHz, and 1 MHz, respectively. In addition, by taking advantage of real time high sensitivity, active phase noise reduction of an RF oscillator is also demonstrated.

## Working Principle and Experimental Setup

**Figure 1:**
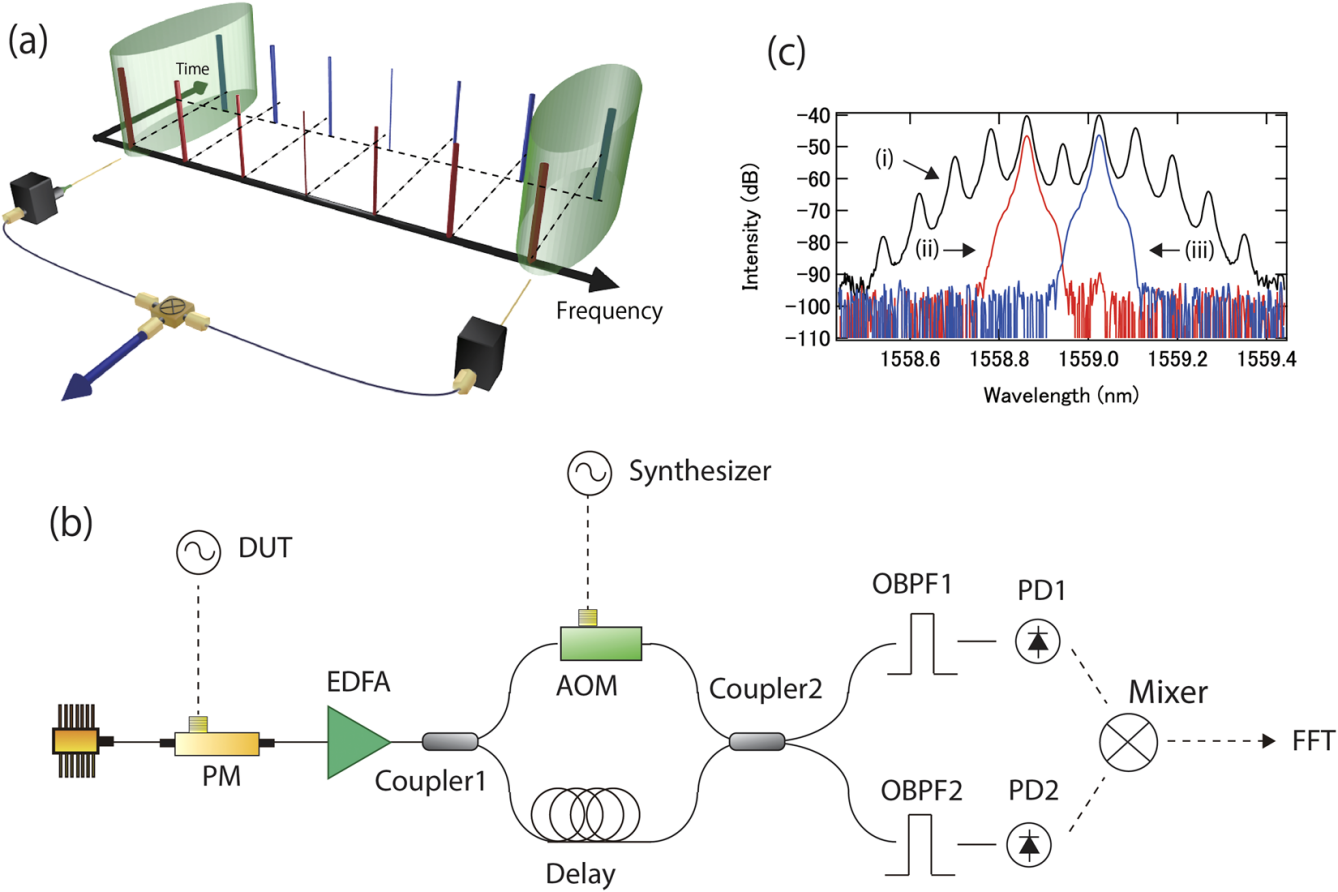
(**a**) Schematic of working principle. Pairs (highlighted by green cylinders) of frequency-shifted (red) and time delayed (blue) EO combs are photo-detected, and mixed. (**b**) Schematic of experimental setup. DUT, device under test; PM, phase modulator; EDFA, Er doped fiber amplifier; AOM, acousto-optic modulator; OBPF, optical bandpass filter; PD, photo detector. (**c**) Optical spectra after the phase modulator (i, black), and optical BPFs (ii, red) and (iii, blue).

Figure 1 shows schematics of the working principle (a) and experimental setup (b) of our method. It is using a “two wavelength delayed self-heterodyne interferometer”^18, 19^. The two﻿ wavelength delayed self-heterodyne interferometer has been originally developed to measure the timing jitter of mode-locked lasers. By using two separate wavelengths, high sensitivity can be obtained. In our method, a mode-locked laser is replaced by an EO comb, which is driven by a DUT. In this case, phase noise of the DUT can be measured with high sensitivity via the use of high-order sidemodes of the EO comb. An EO comb is generated when a cw laser with 1560 nm center wavelength passes a phase modulator. The phase modulator is modulated by a DUT with around 10 GHz or 20 GHz, generating up to +/−10th harmonics in this report. Phase noise of the +/−nth EO comb mode is represented as ${\phi }_{cw}(t)\pm n{\phi }_{DUT}(t)$, where ${\phi }_{cw}(t)$ and ${\phi }_{DUT}(t)$ are phase noise of the cw laser and the DUT in the time domain, respectively. The EO comb is split in two by a 50:50 optical coupler. One arm is time delayed by a photonic delay line (about 100 m or 1 km in this report) (blue), and the other is frequency-shifted by e.g. an AOM (red). After this process, the two EO combs are interfered by a 2 × 2 optical coupler. Two outputs from the optical coupler are optically bandpass filtered to extract only a pair of EO comb modes, e.g. the +/−nth EO comb mode pair. Subsequently, the extracted pairs of EO comb modes are photo-detected. At the photo detectors, beat signals at the AOM frequency are obtained. The signals contain phase noise from the fiber delay line, AOM, cw laser, intensity noise of the cw laser through AM-PM conversion, and DUT. Note that because the frequency of the detected signal is at the heterodyne frequency, i.e. AOM frequency (at ~100 MHz), our method does not require a large bandwidth for photo detection and any of the following RF components, unlike required for the conventional delayed self-homodyne method. By multiplying two signals with an RF mixer at quadrature, the voltage noise power spectrum density (PSD) of the signal from the RF mixer (V_out_(f)^2^) is

1 $${V}_{out}{(f)}^{2}={K}_{1}{(2n)}^{2}{|H(jf)|}^{2}{L}_{DUT}(f).$$

Here, K_1_ is a coefficient which connects phase noise power spectrum density with voltage noise power spectrum density, whereas f, *H*(*jf*), and *L*_*DUT*_(*f*) are Fourier frequency offset, delay transfer function and SSB (single side band) phase noise PSD of the DUT, respectively. At the RF mixer, phase noise of the cw lasers and the AOM is cancelled out, and fiber noise is down-converted from the optical to the DUT frequency. A detailed derivation of (1) is shown in the supplementary section. The above expression is the same as can be derived for the delayed self-homodyne method^16^, except for the ‘(2n)^2^’ factor. Because of the (2n)^2^ factor, the sensitivity is enhanced by 20 × log(2n) dB until the sensitivity reaches the limit set by the fiber delay line. In other words, phase noise of the DUT is enhanced, while contributions to phase noise from background noise (including electronic and shot noise), residual cw phase noise and AM-PM conversion through the system remain the same. More details are discussed in the following sections.

## Experimental Result

### Phase noise measurement

Figure 1(c) shows an example of the optical spectrum of the EO comb after the phase modulator. The RF power for the phase modulator is adjusted to optimize the +/−1st comb mode power, and the center wavelength of the optical bandpass filter (BPF)s are set at +/−1^st^ comb mode wavelengths. Optical spectra after the optical BPFs are also shown in Fig. 1(c), exhibiting more than 50 dB side-mode suppression.

**Figure 2:**
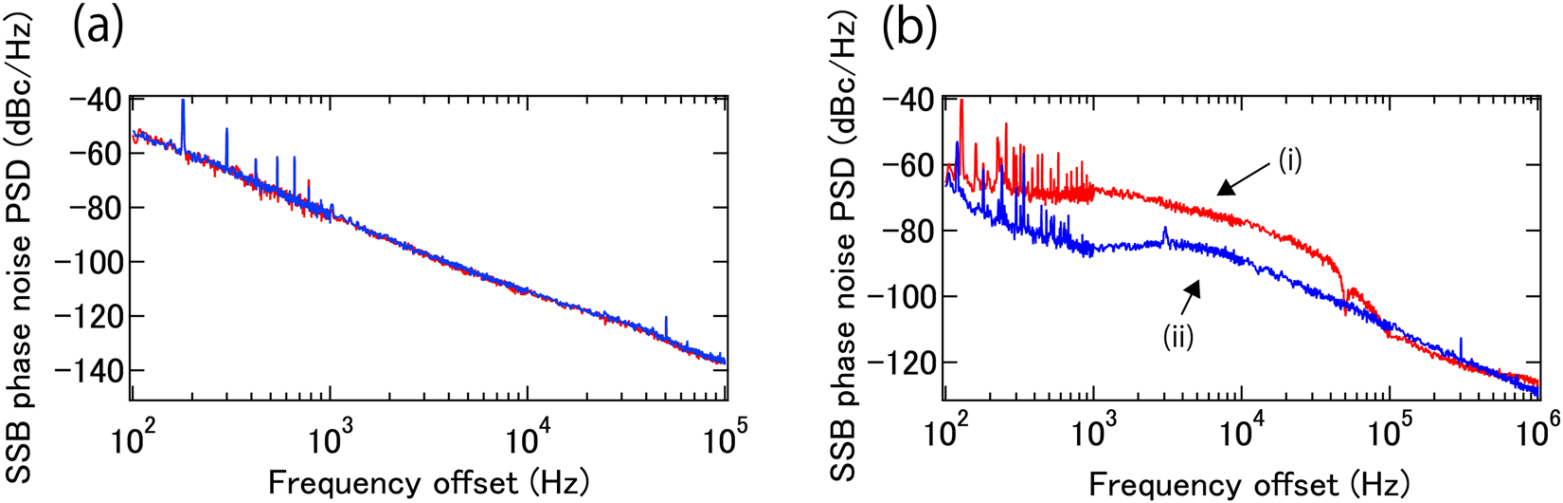
(**a**) SSB phase noise PSD of the 10 GHz DUT measured by the proposed method (red) and conventional delayed self-homodyne method (blue). (**b**) SSB phase noise PSD of the 20 GHz DUTs from WILTRON, 6747A (i, red) and Hewlett-Packard, 8341A (ii, blue).

The phase noise of a DUT (10 GHz carrier frequency) as measured with 1 km fiber delay is shown in Fig. 2(a). As a validation of the obtained phase noise, the conventional delayed self-homodyne method is also implemented. As shown in Fig. 2(a), the phase noise obtained with the two methods has little discrepancy, which means the output from the RF mixer follows the theoretical expression from equation (1). To demonstrate the principle of the present phase noise measurement system for high RF frequencies the phase noise of two DUTs with about 20 GHz carrier frequency is also measured. The result is shown in Fig. 2(b), where 100 m fiber delay is used to extent the frequency offset range to 1 MHz. The experimental setup is the same as required for the measurement for a DUT with 10 GHz carrier frequency, except for changing the RF amplifier before the phase modulator and tuning the bandpass filters to appropriate transmission wavelengths.

### Enhancement of sensitivity

**Figure 3:**
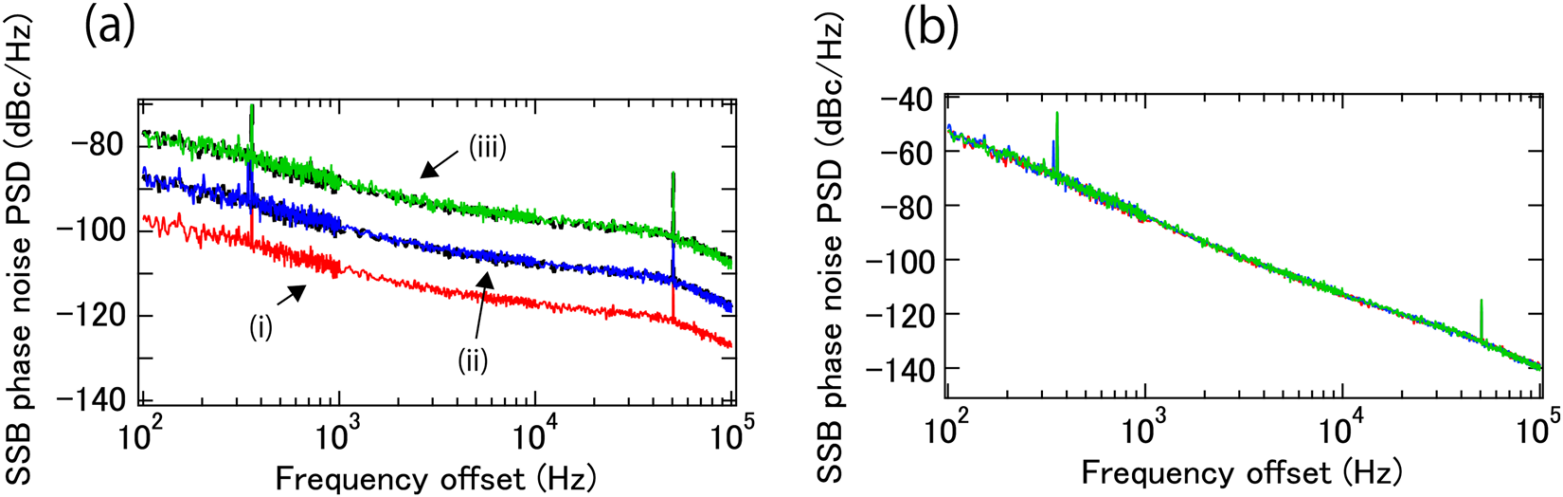
(**a**) SSB phase noise PSD of the mixer output when +/−1st (i, red), +/−3rd (ii, blue), and +/−10th (iii, green) are used. Theoretically expected phase noise for +/−3rd and +/−10th from +/−1st are also shown as dotted black curves. (**b**) SSB phase noise PSD of the DUT estimated from (**a**).

Using the higher side-mode of the EO comb allowed for a convenient demonstration of sensitivity enhancement. Figure 3(a) shows the phase noise PSDs of the signal after the mixer when using the +/−1st, +/−3rd, and +/−10th mode pairs. RF power for the phase modulator is adjusted by an RF attenuator after the DUT to optimize the specified comb modes. The increase of the PSDs follow closely the theoretically expected values (9.5 dB for +/−3rd and 20 dB for +/−10th side-modes). This is further verified in Fig. 3(b), which shows the SSB phase noise PSD of the DUT scaled by the enhancement factor (2n)^2^; as expected, after scaling the traces in Fig. 3(a) related to the different order mode pairs all overlap. Except for fiber delay noise, phase noise magnification of the DUT is useful to minimize unavoidable noise contributions from other noises sources [e.g. background noise (either electronic noise or shot noise), residual phase noise of the cw laser because of imperfect cancellation, or unwanted AM-PM conversion through the system] to enhance the sensitivity, because only phase noise of the DUT is magnified, while other noise contributions remain the same. As discussed in the next section and supplementary section, the sensitivity when using the +/−10th sidemodes is improved by 20 dB compared with using the +/−1st sidemodes.

### Estimation of the sensitivity

We considered four kinds of noise, which limit the sensitivity; (1) background noise, (2) fiber delay noise, (3) residual phase noise of the cw laser, and (4) AM – PM conversion noise. Background noise comes from either shot noise or electronic noise.

For the estimation of background noise, we input the same signals as in the actual phase noise measurement, but without the fiber delay. In this case, phase noise of the DUT and fiber delay don’t show up after the mixer, because the delay transfer function has no contribution at least up to 1 MHz frequency offset. By dividing the phase noise PSD of the output from the mixer by the delay transfer function for 100 m and 1 km fiber with the enhancement factor ((2*n*)^2^|*H*_100*m*_(*jf*)|^2^)or (2*n*)^2^|*H*_1*km*_(*jf*)|^2^)), the sensitivity limit by the background noise can be estimated.

For the estimation of fiber delay noise, we use a phase noise PSD of the 0th mode of the EO comb after the photo detector(${L}_{PD}(f)$), which can be expressed as ${L}_{PD}(f)={(2\pi {f}_{c})}^{2}{L}_{fiber}(f)+|H{(jf)}^{2}|{L}_{cw}(f)$, where L_fiber_(f) and L_cw_ (f) are phase noise PSD of the fiber delay and cw laser, respectively. Note that L_fiber_(f) is defined as the phase noise of the fiber delay around DC. When using a low phase noise cw laser, maximum fiber noise can be estimated, although the estimation is very likely limited by the phase noise of the cw laser. As shown in Fig. S1(a). in the supplementary section, maximum fiber noise up to 100 Hz can be estimated. Above 100 Hz, the phase noise of the cw laser is higher than fiber noise. By extrapolation, i.e. assuming fiber noise is proportional to 1/f^2^ ^20, 21^, we can estimate the fiber noise at frequencies above 100 Hz. Once fiber noise at the optical frequency is estimated, the fiber noise at the DUT frequency can be estimated via the down-conversion factor ${(\frac{{f}_{c}}{{f}_{DUT}})}^{2}$. The sensitivity limit governed by fiber delay noise is then obtained via division by ${|{H}_{100m}(jf)|}^{2}\,{\rm{or}}\,{|{H}_{1km}(jf)|}^{2}$. More details are discussed in the supplementary section.

Residual phase noise of the cw laser, although suppressed in the ideal case, may be observed experimentally because of imperfect cancellation of the noise at the mixer. To characterize the cancellation factor, another phase modulator is inserted after the cw laser to apply a known amount of phase noise. By watching the signal at the modulation frequency after the mixer, the cancellation factor can be experimentally measured. In the best case, the cancellation factor is about −90 dB, although we found, for the best cancellation factor, the path length difference after coupler 2 and the relative phase between the two inputs to the mixer need to be optimized. The reason is not clear yet, and we will investigate more. By dividing the phase noise of the cw laser by the cancellation factor with the appropriate enhancement factor, the sensitivity limit from the residual phase noise of the cw laser can be estimated. The sensitivity limit due to phase noise of the cw laser improves with enhancement factor. Here, phase noise of the cw laser is measured by using a signal after the PD. More details are discussed in the supplementary section.

To investigate the AM – PM conversion noise contributions to the measured phase noise, we compared the intensity noise before the PD to the measured phase noise. The intensity noise before the PD originates at two points; (i) before coupler 1 and (ii) before coupler 2. For (i), we added AM noise by modulating the pump current of the EDFA. For (ii), the RF power delivered to the AOM is modulated. The added intensity noise is measured after the BPF, and compared with the signal after the mixer. AM – PM conversion through the system is about − 20 dB for both (i) and (ii). Then, by dividing by ${(2n)}^{2}{|{H}_{100m}(jf)|}^{2}\,{\rm{or}}\,{(2n)}^{2}{|{H}_{1km}(jf)|}^{2}$, the sensitivity limit by AM – PM conversion can be estimated. In this case, the sensitivity limit governed by AM – PM conversion also improves with enhancement factor. More details are discussed in the supplementary section.

**Figure 4:**
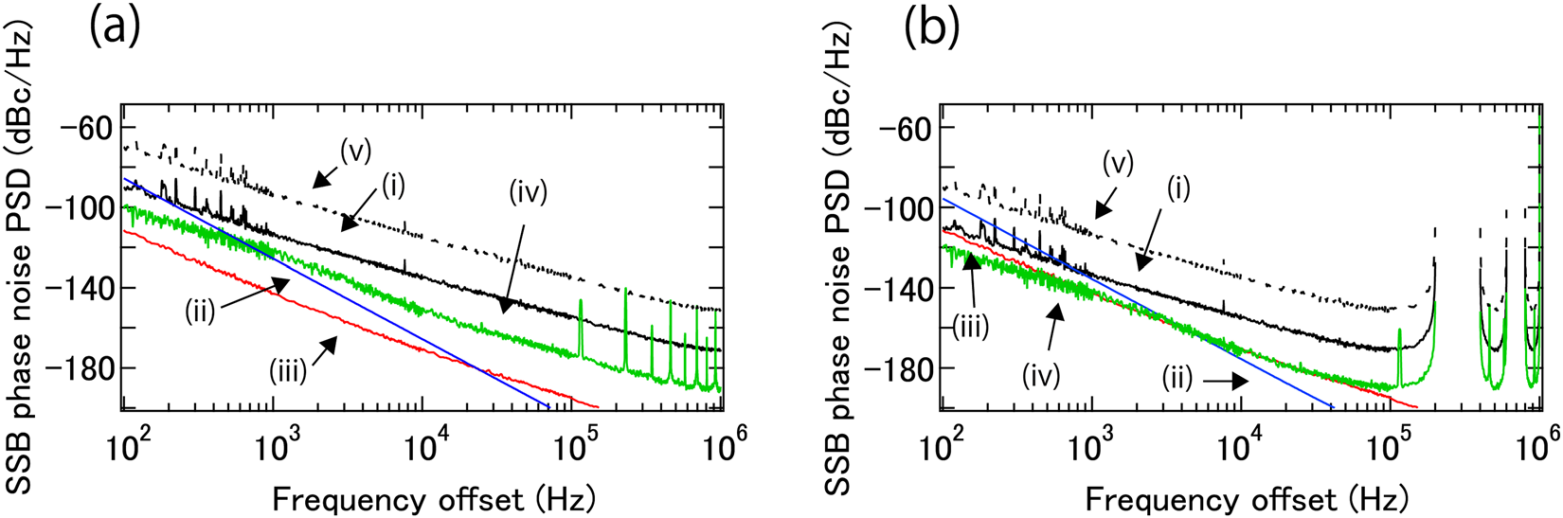
Sensitivity of the phase noise measurement for a 100 m fiber (**a**) and 1 km fiber delay (**b**) for +/−10 th harmonics. (i, black), (ii, blue), (iii, red), and (iv, green) curves are sensitivity limits from background noise, fiber noise, cw laser noise, and intensity noise before PDs for the use of +/−10th order sidemodes, respectively. (v, dotted black) curves are sensitivity limits from background noise for the use of +/−1st order sidemodes.

Figure 4(a) and (b) summarize the sensitivity limited by these four noise terms for both 100 m and 1 km fiber delay when +/−10th harmonics are used. The sensitivity limits by background noise when using +/−1st harmonics are also shown. The enhancement obtained from the use of higher order sidemodes of the EO comb improves the sensitivity in terms of background noise, residual phase noise of the cw laser, and AM – PM conversion noise; only the fiber nose is unaffected via the use of harmonics. For both 100 m and 1 km fiber delay lengths, the main limitation is either background noise or fiber delay noise. Note that estimation of the fiber noise is very conservative, and actual fiber noise is very likely much smaller. When low order sidemodes of the EO comb are used, background noise is the main limitation. When higher order sidemodes of the EO comb are utilized, the sensitivity reaches the fiber noise limits at low frequency offset. At high frequency offset, background noise still limits the sensitivity, indicating the use of more than +/−10th harmonics should enable even higher sensitivity. In summary, when 1 km (100 m) fiber delay length in conjunction with +/−10th harmonics are used, the sensitivity is −133 (−113) dBc/Hz, −155 (−135) dBc/Hz, −170 (−154) dBc/Hz and n.a. (−171) dBc/Hz at 1 kHz, 10 kHz, 100 kHz and 1 MHz Fourier offset frequency for a 10 GHz carrier.

### Phase noise reduction of RF oscillator

**Figure 5:**
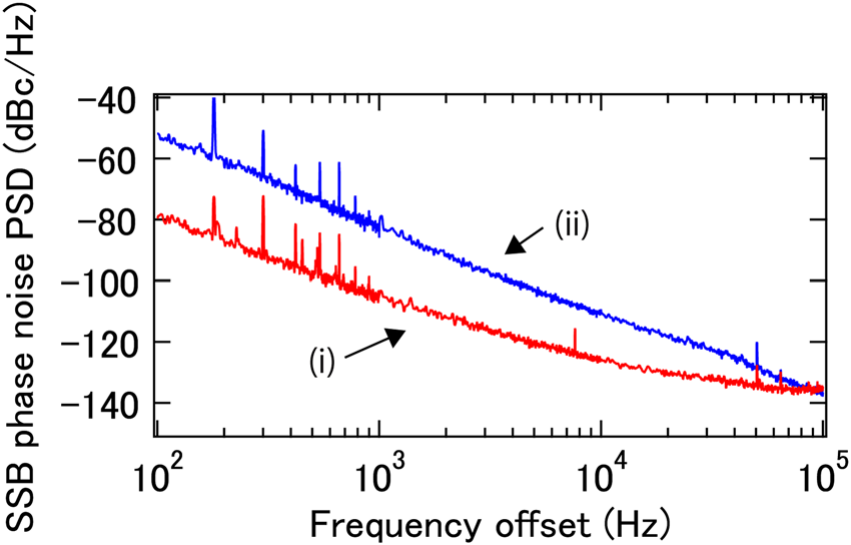
SSB phase noise PSD of the DUT with (i, red) and without (ii, blue) feedback measured via an out-of-loop measurement.

As shown in the previous section, the proposed method excels with high sensitivity in real time, without resorting to cross correlation. Because it is a real time method, the signal after the mixer can be used to suppress the phase noise of the DUT by feeding back to the DUT. With 1 km fiber delay, phase noise suppression as performed on the 10 GHz DUT (which is the same DUT as used for Figs 2(a) and 3) is shown in Fig. 5. Here, the +/−1st order sidemodes are used, not higher order sidemodes, because intrinsic phase noise of the DUT with limited feedback bandwidth determines the achievable phase noise. In this experiment, the suppressed phase noise is measured out-of-loop, based on a conventional delayed self-homodyne method with a 1 km fiber delay. Phase noise reduction up to 100 kHz is observed. The feedback bandwidth is limited by the fiber length. The obtained phase noise is limited by the sensitivity of the out-of-loop measurement. Note that because the proposed method does not rely on electronic and O/E components with high bandwidth, the phase noise reduction demonstrated here is compatible with microwave carrier frequencies limited only by the modulation bandwidth of the EOM, which can reach up to 100 GHz.

## Discussion and Conclusion

As shown in Fig. 4, the sensitivity of the present system can ultimately reach the fiber noise limit when using higher order sidemodes of the EO comb. However, fundamental thermally limited fiber noise is far below our fiber noise^20, 21^, which indicates a well designed enclosure can improve the fiber noise. Again note that the estimation of our fiber noise is very conservative, and the fiber noise is very likely much smaller. In fact, the fiber noise reported by Dong *et al*., is close to the thermal noise limit^22^. Another way to reduce fiber noise is to actively stabilize the fiber to an ultra-low phase noise cw laser. This additional feedback loop can be easily added to the setup. At the PD (up-stream from the mixer), we can access a signal containing both fiber noise and phase noise of the cw laser, which can be used as an error signal to stabilize the fiber to the cw laser^22–24^.

As shown in Fig. 2(b), our method can be easily extended to high frequency carriers. The sensitivity degrades compared with lower frequency carriers, if fiber noise is the main limiting factor determining sensitivity. The sensitivity degrades by 20 × log(f_DUT_/10 GHz) [dB], compared with the sensitivity for a 10 GHz carrier. This degradation is much slower than exhibited by present-day phase noise analyzers based on microwave technology.

A long fiber has higher sensitivity, sacrificing the measurable frequency offset range. When the sensitivity is not limited by the fiber, the sensitivity is proportional to 1/L^2^ (i.e.1/|*H*_*L*_(*jf*)^2^|). On the other hand, when the fiber noise limits the sensitivity, the sensitivity is proportional to 1/L (i.e.*L*_*fiber*_(*f*)/|*H*_*L*_(*jf*)^2^|). Here L_fiber_(f) is fiber noise and is proportional to L. Measurable frequency offset range is limited up to *c*/*nL* (n is refractive index of the fiber) because of the null frequency of the delay transfer function. As shown in the next paragraph, nonlinear effects during propagation through the fiber need to be taken care of when more than 10 km fiber is used.

To use higher order modes, EO combs with high average power need to propagate through the delay fiber, which may cause degradation of the sensitivity via nonlinear effects such as stimulated Brillouin and Rayleigh scattering because of excess intensity noise and phase noise. Although stimulated Brillouin scattering scatters light in the backward direction, intensity noise of the forward-propagating beam becomes worse when the “power” of the propagating light approaches the threshold for stimulated Brillouin scattering. Since the threshold is determined by the “power per comb mode”, even when increasing the average power with the use of higher-order comb modes, the power per EO comb mode can stay the same and not lead to the onset of stimulated Brillouin scattering. In fact, we did not observe any excess intensity noise at elevated power levels. Stimulated Rayleigh scattering may also cause excess phase noise by co-propagating with the EO comb. We measured the phase noise PSD of the signal at the PD while changing the input power to the fiber delay. No excess phase noise was observed at least up to 90 mW mode power when using a 1 km fiber delay line. More details are discussed in the supplementary section. Because a power of 90 mW far exceeds the typical saturation level of PDs, we expect neither stimulated Brillouin nor Rayleigh scattering will affect sensitivity. Moreover, our phase noise analyzer is compatible with the use of a 10 km fiber delay length, allowing for further improvement in sensitivity at or below a frequency offset of 10 kHz. Because the threshold of stimulated Brillouin scattering is proportional to the inverse of fiber length, no excess noise is expected up to power levels of 9 mW for a 10 km fiber.

To generate up to +/−10th harmonics with optimum mode power, about 33 dBm RF power is required for our phase modulator, so that an RF amplifier with up to +35 dBm needs to be installed between the DUT and the phase modulator. The RF amplifier may have larger phase noise than the estimated sensitivity shown in Fig. 4. However, selecting an adequate RF amplifier in terms of the noise figure, gain, and output power will enable additive phase noise below −170 dBc/Hz above 10 kHz frequency offset. To reduce the required RF power, EDFA with higher output can be used, instead of optimizing specified harmonic sidebands. Alternatively, nonlinear spectrum broadening via four wave mixing can also be implemented^25^. In addition, phase modulators with ultra-low V_π_ (the voltage required to cause π phase change) have been developed^26^.

Regarding phase noise reduction via feedback, the ultimate limitation is the sensitivity of our proposed method, although the results shown in Fig. 5 are worse than expected from sensitivity. Currently, the obtained phase noise is limited by the sensitivity of the out-of-loop measurement. A more critical limit is the feedback bandwidth, i.e. feedback gain. Improving the feedback bandwidth is difficult since it is limited by the fiber length. To obtain lower phase noise, we need to use an RF oscillator with intrinsically low phase noise. By using intrinsically low phase noise RF oscillators, −100 dBc/Hz at close to carrier frequency is feasible by carefully designing an environmental isolation system such as ref. 22. Note that using intrinsically low phase noise RF oscillators is not very effective in conjunction with other methods for active phase noise suppression, for example based on the use of high harmonics of an EO comb, since the sensitivity is not as good as the present method even when using sidemodes of an EO comb up to a harmonic order of 100^10, 27^.

The components for the photonic subsystem required for the present phase noise analyzer are entirely all-fiber based, and there is no need for any sophisticated modules such as ultra-low noise optical frequency combs and cw lasers stabilized to ultra low noise cavities. This is a great benefit of this method compared to alternative ultra-low noise “fixed” frequency microwave generation and characterization systems^7, 28^.

In conclusion, we have demonstrated a low phase noise measurement system based on a two wavelength, delayed self heterodyne interferometer, in which high order sidemodes of an EO comb are utilized for sensitivity enhancement, resulting in a sensitivity of −133 (−113) dBc/Hz, −155 (−135) dBc/Hz, −170 (−154) dBc/Hz and n.a. (−171) dBc/Hz at 1 kHz, 10 kHz, 100 kHz and 1 MHz Fourier offset frequency for a 10 GHz carrier, respectively, via 1 km (100 m) fiber. The method can be easily extended to higher frequency DUTs, while preserving high sensitivity, because the method does not require large bandwidth photo detectors nor high frequency RF components. Moreover, because the phase noise measurement system has real time sensing capability, the measured signal was efficiently implemented to reduce the phase noise of a DUT microwave oscillator. We believe the present system will be invaluable for further advancing the dissemination of microwave photonics in high frequency RF technology. It greatly simplifies the characterization of ultra-low noise microwaves and offers exciting opportunities for many emerging applications that rely on readily available ultra-low phase noise microwaves.

## Method

### Experimental setup for the phase noise analyzer

We employed a cw laser with a specified linewidth of 1.8 kHz, 10 mW output power, and 1558.94 nm wavelength (RIO ORION laser module). The 10 GHz DUT (INWAVE, DRO-10010) used for Figs 2(a), 3 and 5 produces about +10 dBm output power and has a modulation bandwidth of more than 500 kHz. In this report, up to +/−10th harmonics are exploited. To generate +/−10 th harmonics with optimum mode power, 3.7π rad phase modulation is required, which corresponds to about 33 dBm for our phase modulator. Because of this, an RF amplifier with up to +35 dBm output power needs to be installed between the DUT and the phase modulator. After generation of the EO comb, the EO comb is amplified by an EDFA. The output power from the EDFA is 32 mW, 59 mW, and 115 mW when used in conjunction with the +/−1st, +/−3rd, and +/−10th EO comb modes, respectively, which generate signals of about −26 dBm RF power at the photo detectors. The amplified EO comb is split in two by a 50:50 optical coupler. In one arm, the EO comb is frequency shifted by about +80 MHz via an AOM. In the other arm, the EO comb passes through another AOM, configured for a frequency shift of about −80 MHz, and the photonic fiber delay line(we use about 100 m and 1 km fiber). In principle, the implementation of only one AOM frequency shifter should be sufficient. However, we found that without the additional AOM, spurious signals originating from the non-diffracted light transmission through the AOM (at around −60 dB) cause detrimental effects to the system. The inclusion of the second AOM effectively ‘doubles’ the extinction ratio between diffracted and non-diffracted light transmission through the AOMs to around −120 dB. The fiber delay line is put in a box with 5 mm thickness aluminium with a sound absorbing foam inside. No vibration isolation board from the ground is installed. After both arms are combined with a 4 port optical coupler with splitting ratio of 50:50, each output is directed through optical bandpass filters (Yenista Optics, XTM-50 and Alnair Labs, BVF-300). The central wavelength of the optical bandpass filters is tunable, and the FWHM bandwidth is less than 10 GHz. The bandpass filtered EO comb mode pairs are photo-detected with commercially available photo detectors (EOT, ET-3010). After photo detection, RF signals with 160 MHz carrier frequency are RF amplified and filtered, and electronically mixed in an RF mixer. Quadrature at the RF mixer is ensured by adjusting optical delay or, for experimental convenience, the DUT frequency. The quadrature point cannot keep for long term because of temperature drift of the fiber delay. If continuous long term operation is necessary, to compensate the drift, output from the RF mixer can be used as an error signal and fed-back to a voltage-adjustable optical delay.

### Calibration of phase noise

The voltage noise PSD of the signal (V_out_(f)^2^) after the mixer is measured with a signal analyzer (Hewlett-Packard, 89410A), which can be represented as ${V}_{out}{(f)}^{2}={K}_{1}{(2n)}^{2}{|H(jf)|}^{2}{L}_{DUT}{(f)}^{2}$. The delay transfer function can be expressed as ${|H(jf)|}^{2}=4\,{\sin }^{2}(\pi \tau f)$ ^16^. The fiber delay τ can be measured experimentally by watching the null point of the signal after the PDs. To evaluate the coefficient K_1_, a single tone phase modulation at modulation frequency (f_m_) is added to the DUT. The added phase noise (L_DUT_(f_m_)) is then measured by an electronic spectrum analyzer. From the signal after the mixer, V_out_(f_m_)^2^ is measured. Then, K_1_ is estimated as ${K}_{1}=\frac{{V}_{out}{({f}_{m})}^{2}}{{(2n)}^{2}{|H(j{f}_{m})|}^{2}{L}_{DUT}({f}_{m})}$. Once K_1_ is estimated, L_DUT_(f) can be estimated from V_out_(f)^2^.

### Experimental setup for conventional delayed self-homodyne phase noise analyzer

For the conventional delayed self-homodyne method, another cw laser (OEwaves) is used. The output from the cw laser is intensity modulated by an intensity modulator. Then, the modulated signal is amplified by an EDFA, followed by a 1 km fiber delay and a high bandwidth (>10 GHz) photo detector (Discovery semiconductors, DSC40S). The signal after the PD is about −4 dBm, and input to an RF mixer with a local input from the DUT after passing an RF amplifier. The calibration procedure is the same as described above.

## Electronic supplementary material

Supplementary information
