# Supplementary material for: Electro-optic comb based real time ultra-high sensitivity phase noise measurement system for high frequency microwaves
Source: Sci Rep. 2017 Jun 6;7:2847. doi: 10.1038/s41598-017-03049-5 (PMC5460219; doi:10.1038/s41598-017-03049-5)
Supplement: Supplementary file 1 — Supplementary information [file 41598_2017_3049_MOESM1_ESM.pdf]

Supplementary Information

Electro-optic comb based real time ultra-high sensitivity phase noise measurement system for high frequency microwaves

Naoya Kuse[1]*, and Martin E. Fermann[2]

[1] *IMRA America Inc., Boulder Research Labs, 1551 South Sunset St, Suite C, Longmont, CO, 80501, USA*

[2] *IMRA America Inc., 1044 Woodridge Ave, Ann Arbor, MI, 48105, USA*

*e-mail: nkuse@imra.com

1. Mathematical expression of the phase noise analyzer

The signals observed at the PDs ($i_{\pm n}(t)$) in the time domain can be represented as

$$i_{\pm n}(t) \propto \cos(2\pi f_{AO}t + 2\pi\left(f_c \pm nf_{DUT}\right)\tau + \varphi_{AO}(t) + \varphi_{cw}(t) - \varphi_{cw}(t-\tau) \pm n\left(\varphi_{DUT}(t) - \varphi_{DUT}(t-\tau)\right)) \quad \text{(S1).}$$

Here, f_{AO}, f_c, and f_{DUT} are AOM frequency, cw laser frequency, and DUT frequency, respectively. φ_{AO}, φ_{cw}, and φ_{DUT} are phase noise of the AOM, cw laser and DUT, respectively. τ is the delay time in the self-heterodyne interferometer. By multiplying the two signals by an RF mixer, the output from the RF mixer in the time domain is obtained as

$$V_{out}(t) \propto \cos\left(2\pi \cdot 2nf_{DUT}\tau + 2n\left(\varphi_{DUT}(t) - \varphi_{DUT}(t-\tau)\right)\right) \quad \text{(S2).}$$

At the RF mixer phase noise from the cw laser and AOM is cancelled out, and fiber noise is down-converted from the optical to the RF frequency domain. Once $2\pi \cdot 2nf_{DUT}\tau$ is set to 90 degrees, V_{out} can be approximated as,

$$V_{out}(t) \propto 2n\left(\varphi_{DUT}(t) - \varphi_{DUT}(t-\tau)\right) \quad \text{(S3).}$$

This signal is Fourier transformed with a signal analyzer, producing a voltage noise PSD of ($V_{out}(f)^2$) as,

$$V_{out}(f)^2 = K_1 \cdot (2n)^2 \left|H(jf)\right|^2 L_{DUT}(f) \quad \text{(S4).}$$

Here, f, $H(jf)$, and $L_{DUT}(f)$ are the offset from the microwave carrier frequency, the delay transfer function, and the SSB (single side band) phase noise PSD of the DUT, respectively. The delay transfer function is

$$\left|H(jf)\right|^2 = 4\sin^2\left(\pi\tau f\right) \quad \text{(S5).}$$

The delay transfer function has null frequencies at 1/τ as shown in Fig. S1. The null frequencies for 100 m and 1 km fiber are 200 kHz and 2 MHz, respectively. Measurable frequency offset is limited up to the null frequency. For safety, we used results up to half of the null frequency.

Experimentally, there are other noise contribution to equation (S4). When we consider fiber noise, background noise, residual phase noise from cw laser, and AM-PM conversion of the light through the system, equation (S4) can be expressed as

$$V_{out}(f)^2 = K_1[(2n)^2 \left|H(jf)\right|^2 L_{DUT}(f) + \left(2\pi \cdot 2nf_{DUT}\right)^2 L_{fiber}(f) + L_{background}(f) + C_{PM}\left|H(jf)\right|^2 L_{cw}(f) + C_{AM} L'_{cw}(f)] \quad \text{(S6).}$$

Here, $L_{fiber}(f)$, $L_{background}(f)$ and $L_{cw}(f)$ are respectively, fiber delay phase noise (defined at DC), background noise including electrical components and shot noise, and phase noise of the cw laser. $L'_{cw}(f)$ is the intensity noise of the light before the PD. C_{PM} is the cancellation factor of the phase noise of the cw laser at the mixer, and C_{AM} is the AM-PM conversion factor of the light through the system upstream of the PD.

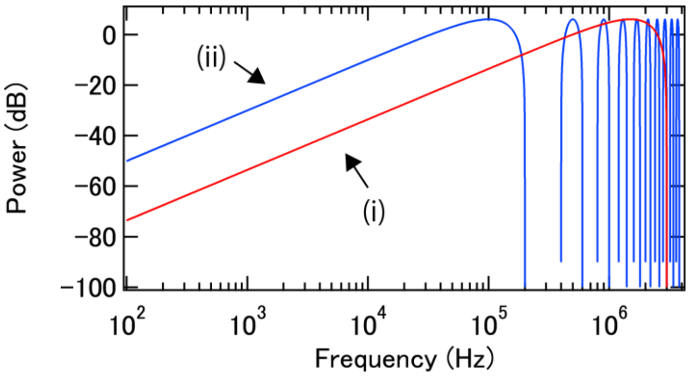

Figure S1. Delay transfer function ($|H(jf)|^2$) for 100 m (i, red) and 1 km (ii, blue).

2. Limitation by fiber noise

Because fiber noise is one of the critical factors determining the sensitivity especially for low frequency offsets, fiber noise needs to be characterized. When the 0th order mode of the EO comb is detected, the voltage noise PSD after the PD ($V_{PD}(f)^2$) can be represented as,

$$V_{PD}(f)^2 = K_2\left((2\pi f_c)^2 L_{fiber}(f) + |H(jf)|^2 L_{cw}(f)\right) \qquad \text{(S7)}$$

Here, K_2 is the SSB phase noise of the cw laser and the conversion coefficient for conversion from voltage noise PSD to phase noise PSD, respectively. To evaluate the coefficient K_2, a known single tone phase noise ($L_{cw}(f_m)$) is added to the cw laser, and the resulting voltage noise at the modulation frequency ($V_{PD}(f_m)$) is measured. Experimentally, the phase modulation is added to the cw laser by using another phase modulator after the cw laser. At a Fourier frequency offset, where $|H(jf_m)|^2 L_{cw}(f_m)$ is higher than $(2\pi f_c)^2 L_{fiber}(f_m)$, the coefficient K_2 is estimated as

$K_2 = \frac{V_{PD}(f_m)^2}{|H(jf_m)|^2 L_{cw}(f_m)}$. By using this, the phase noise PSD of the signal at the PD can be obtained as shown in Fig. S2(a). The maximum fiber noise is estimated by fitting below 100 Hz frequency offset. Note that it is very likely that estimation is limited by the phase noise of the cw laser. Actually, thermal noise limit [S1, S2] is much smaller than estimated fiber noise. By extrapolation of the fiber noise, while assuming the fiber noise is proportional to $1/f^2$, fiber noise in the optical domain ($(2\pi f_c)^2 L_{fiber}(f)$) can be estimated as < - $20/f^2$ dBc/Hz/km. Once the fiber noise is estimated, the sensitivity limit from the fiber noise ($\varphi_{limit,fiber}(f)^2$) can be estimated as,

$$\varphi_{limit,fiber}(f)^2 = \frac{(2\pi \cdot 2nf_{DUT})^2}{(2n)^2 |H(jf)|^2} \cdot L_{fiber}(f) = \frac{(2\pi f_{DUT})^2}{|H(jf)|^2} \cdot L_{fiber}(f) \qquad \text{(S8)}$$

$L_{fiber}(f)$ is proportional to fiber length. On the other hand, the delay transfer function ($|H(jf)|^2$) is proportional to the square of fiber length, so that the sensitivity limit from the fiber noise ($\varphi_{limit,fiber}(f)^2$) is inversely proportional to fiber length. Figure S2(b) shows the sensitivity limit from fiber noise.

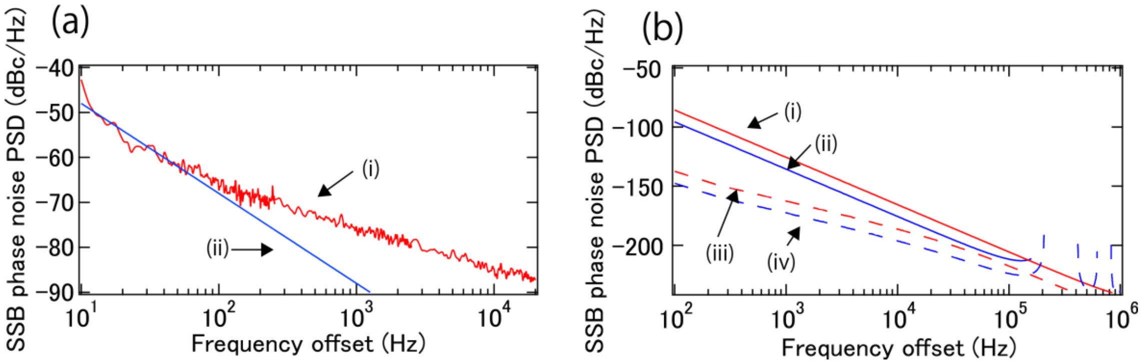

Figure S2. (a) SSB phase noise PSD at the output from the PD (i, red) when 500 m fiber is used. The extrapolated noise contribution from fiber noise (- $25/f^2$ dBc/Hz/km) is also shown (ii, blue). (b) Sensitivity limit due to fiber noise for 10 GHz carrier for 100 m (i, red) and 1 km (ii, blue) fiber estimated from (a). Thermal noise limit for 10 GHz carrier for 100 m (iii, dotted red) and 1 km (iv, dotted blue) fiber.

3. Limitation by residual of cw phase noise

Although, ideally, phase noise of the cw laser is cancelled at the mixer, experimentally, residual cw phase noise can be observed. In this section, we estimate the cancellation factor and resulting sensitivity limit arising from residual cw phase noise. In the presence of residual cw phase noise cancellation, the voltage noise PSD of the output from the mixer can be represented as,

$$V_{out}(f)^2 = K_1\left((2n)^2\left|H(jf)\right|^2 L_{DUT}(f) + C_{PM}\left|H(jf)\right|^2 \cdot L_{cw}(f)\right) \quad \text{(S9)}$$

Here, C_{PM} is the cancellation factor coefficient. As describe in the method section, the coefficient K_1 can be estimated by modulating the DUT with known phase noise, being represented as, $K_1 = \dfrac{V_{out}(f_{m1})^2}{(2n)^2\left|H(jf_{m1})\right|^2 L_{DUT}(f_{m1})}$.

Similarly, by phase modulating the cw laser with known phase noise, the cancellation factor is obtained as below.

$$C_{PM} = \frac{V_{out}(f_{m2})^2}{K_1\left|H(jf_{m2})\right|^2 L_{cw}(f_{m2})} = (2n)^2 \cdot \frac{V_{out}(f_{m2})^2\left|H(jf_{m1})\right|^2 L_{DUT}(f_{m1})}{V_{out}(f_{m1})^2\left|H(jf_{m2})\right|^2 L_{cw}(f_{m2})} \quad \text{(S10)}$$

Once the cancellation factor, C_{PM} is known, the sensitivity limit due to residual cw phase noise ($\varphi_{\lim it,cw}(f)^2$) is estimated as,

$$\varphi_{\lim it,cw}(f)^2 = \frac{C_{PM}\left|H(jf)\right|^2}{(2n)^2\left|H(jf)\right|^2} \cdot L_{cw}(f) = \frac{C_{PM}}{(2n)^2} \cdot L_{cw}(f) \quad \text{(S11).}$$

Experimentally, about -90 dB of cancellation is obtained, resulting in the sensitivity limit from residual of cw phase noise as shown in Fig. S3(b). The sensitivity limit is independent of fiber length and improves with harmonic order. We found experimentally that the path length difference between the upper and lower paths from coupler 2 to the mixer and the relative phase between the two inputs to the mixer need to be optimized for optimum cancellation. The reason is not clear yet, but there may still be detrimental effects from unwanted transmission through the AOMs even with two AOMs.

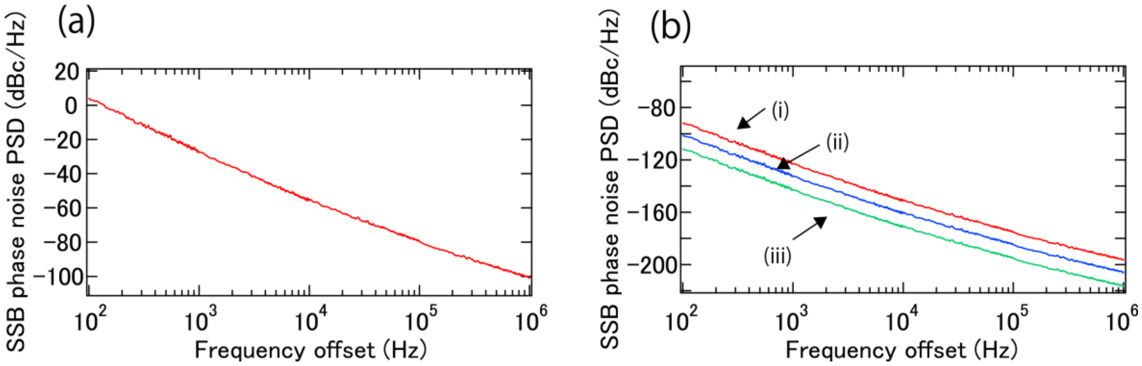

Figure S3. (a) SSB phase noise PSD of the cw laser used in this report. (b) Sensitivity limit due to residual phase noise of the cw laser when +/- 1st (i, red), +/- 3rd (ii, blue), and +/- 10th (iii, green) are used.

4. Limitation by AM-PM conversion

The present system is also susceptible to AM – PM conversion noise, for example from laser intensity noise at the PDs and mixer. To evaluate this influence to the sensitivity limit, we estimated the AM – PM conversion factor of the system. With the AM – PM conversion factor, the voltage noise PSD of the output from the mixer can be represented as,

$$V_{out}(f)^2 = K_1\left((2n)^2\left|H(jf)\right|^2 L_{DUT}(f) + C_{AM} L'_{cw}(f)\right) \qquad \text{(S12)}$$

Here, C_{AM} and $L'_{cw}(f)$ are respectively: the AM – PM conversion factor coefficient and relative intensity noise (RIN) of the light before the PDs. Analogously to the previous sections, the coefficient K_1 can be estimated by phase modulating the DUT with known phase noise, being represented as, $K_1 = \dfrac{V_{out}(f_{m1})^2}{(2n)^2\left|H(jf_{m1})\right|^2 L_{DUT}(f_{m1})}$.

Next, by amplitude-modulating the laser with known amplitude noise, the AM – PM conversion factor can be represented as,

$$C_{AM} = \frac{V_{out}(f_{m2})^2}{K_1 L'_{cw}(f_{m2})} = (2n)^2 \cdot \frac{V_{out}(f_{m2})^2\left|H(jf_{m1})\right|^2 L_{DUT}(f_{m1})}{V_{out}(f_{m1})^2 L'_{cw}(f_{m2})} \qquad \text{(S13)}$$

Once the AM – PM conversion factor, C_{AM} is known, the sensitivity limit due to AM – PM conversion upstream of the PDs is estimated as,

$$\varphi_{\lim it,AM}(f)^2 = \frac{C_{AM}}{(2n)^2 |H(jf)|^2} \cdot L'_{cw}(f) \qquad \text{(S14)}$$

Note there are two places, where amplitude noise is added to the light. One is before splitting (i.e. before coupler 1). The other is after splitting (i.e. before coupler 2). We tried both and found there is little difference in the AM – PM conversion factor. C_{AM} is about – 20 dB. To get the best AM – PM conversion factor, the relative phase between the two inputs to the mixer needs to be optimized. Once the AM – PM conversion factor is obtained, the sensitivity limit due to the AM – PM conversion of the cw laser is estimated as shown in Fig. S4(b) and (c). The sensitivity limit improves with harmonic order.

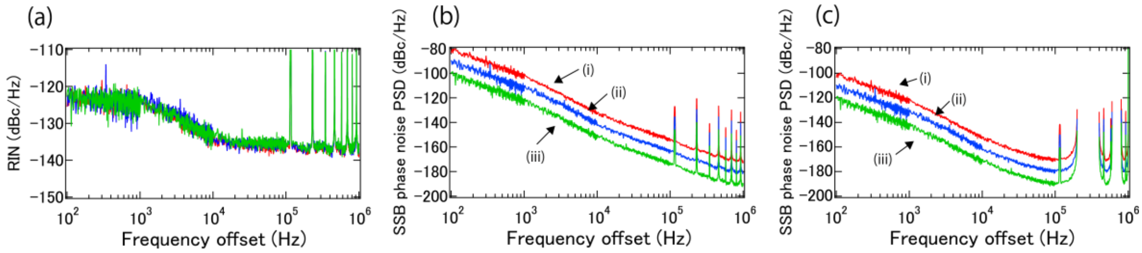

Figure S4. (a) RIN of detected signal measured before the PD for +/- 1st (red), +/- 3rd (blue), and +/- 10th (green). Sensitivity limit for 100 m fiber (b) and 1 km fiber delay. (c) when +/- 1st (i, red), +/- 3rd (ii, blue), and +/- 10th (iii, green) are used.

5. Nonlinear effect to the system

Nonlinear effects such as stimulated Brillouin and Rayleigh scattering are considered in this section. We investigated the effects due to stimulated scatterings to both intensity and phase noise. The experimental setup for intensity noise measurement is shown in Fig. S5(a). We measured the intensity noise of the light after the delay fiber (~ 1 km), while changing the input power, with and without generating an EO comb. In this experiment, the pump current to the EDFA is fixed, and the input power is changed with an optical attenuator. Without generating the EO comb, the intensity noise is significantly degraded for input powers of more than 90 mW as shown in Fig. S5(c). With an EO comb with an output power set to be largest for + / - 3rd sidemodes, no excess intensity noise was observed as shown in Fig. S5(d), indicating that the threshold of stimulated Brillouin scattering is determined by the power per comb modes. 90 mW per comb mode power is more than the saturation power of the PD. Therefore, stimulated Brillouin scattering plays no roles in terms of excess intensity

noise. To check for any excess phase noise from either stimulated Brillouin or Rayleigh scattering, we measured the phase noise PSD of the signal at the PD, while changing the input power. The experimental setup is shown in Fig. S5(b). Even without generating an EO comb, there is no excess phase noise as shown in Fig. S5(e). From these results, we expect increasing the average power of the EO comb to enable the use of higher order side-modes or the utilization of longer fibers are both permissible options for further enhancement of sensitivity of the present phase noise analyzer.

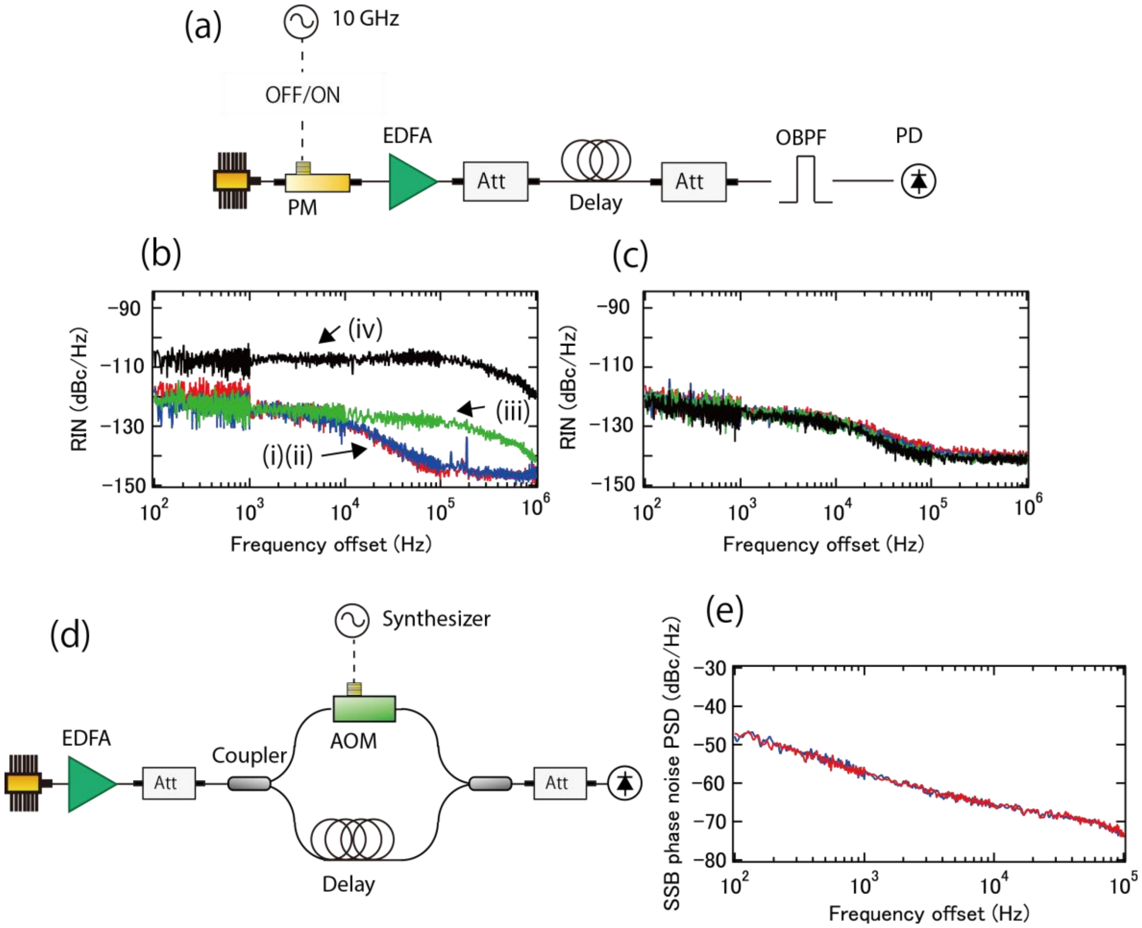

Figure S5. (a) Schematic of experimental setup to check for excess intensity noise. Att, attenuator. Intensity noise of the light before the PD without EO comb (b) and with EO comb (-3rd mode is filtered) (c) for 30 mW (i, red), 70 mW (ii, blue), 90 mW (iii, green) and 110 mW (iv, black). (d) Schematic of experimental setup to check for excess intensity noise. (e) SSB phase noise PSD of the heterodyned signal for 10 mW (red) and 90 mW (blue).

References

[S1] K. H. Wanser, “Fundamental phase noise limit in optical fibres due to temperature fluctuations,” Electron. Lett. 28(1), 53-54 (1992).

[S2] L. Z. Duan, “Intrinsic thermal noise of optical fibres due to mechanical dissipation,”
Electron. Lett. 46(22), 1515-1516 (2010).
